# Apelin receptor homodimer-oligomers revealed by single-molecule imaging and novel G protein-dependent signaling

**DOI:** 10.1038/srep40335

**Published:** 2017-01-16

**Authors:** Xin Cai, Bo Bai, Rumin Zhang, Chunmei Wang, Jing Chen

**Affiliations:** 1 Department of Physiology, School of Medicine, Shandong University, Jinan, Shandong, 250012 P.R. China; 2 Neurobiology Institute, Jining Medical University, Jining, Shandong, 272067 P.R. China; 3 Division of Biomedical Sciences, Warwick Medical School, University of Warwick, Coventry, CV4 7AL, UK

## Abstract

The apelin receptor (APJ) belongs to family A of the G protein-coupled receptors (GPCRs) and is a potential pharmacotherapeutic target for heart failure, hypertension, and other cardiovascular diseases. There is evidence APJ heterodimerizes with other GPCRs; however, the existence of APJ homodimers and oligomers remains to be investigated. Here, we measured APJ monomer-homodimer-oligomer interconversion by monitoring APJ dynamically on cells and compared their proportions, spatial arrangement, and mobility using total internal reflection fluorescence microscopy, resonance energy transfer, and proximity biotinylation. In cells with <0.3 receptor particles/μm^2^, approximately 60% of APJ molecules were present as dimers or oligomers. APJ dimers were present on the cell surface in a dynamic equilibrium with constant formation and dissociation of receptor complexes. Furthermore, we applied interference peptides and MALDI-TOF mass spectrometry to confirm APJ homo-dimer and explore the dimer-interfaces. Peptides corresponding to transmembrane domain (TMD)1, 2, 3, and 4, but not TMD5, 6, and 7, disrupted APJ dimerization. APJ mutants in TMD1 and TMD2 also decreased bioluminescence resonance energy transfer of APJ dimer. APJ dimerization resulted in novel functional characteristics, such as a distinct G-protein binding profile and cell responses after agonist stimulation. Thus, dimerization may serve as a unique mechanism for fine-tuning APJ-mediated functions.

The apelin receptor (APJ) is a member of the family A of G-protein-coupled receptors (GPCRs) and is involved in range of physiological and pathological functions including fluid homeostasis, anxiety, and depression, as well as cardiovascular and metabolic disorders^1^,^2^,^3^. APJ was first identified as an orphan GPCR with closest identity to the angiotensin II (Ang II) receptor. In 1998, the peptide apelin was isolated from bovine stomach extracts as the endogenous ligand of APJ. Subsequently, it was found that apelin could be hydrolyzed by angiotensin-converting enzyme 2 (ACE2) with high catalytic efficiency. Furthermore, the apelin-APJ system plays important roles in cardiovascular homeostasis^4^,^5^,^6^. Initially, APJ was classically described as a monomeric transmembrane receptor that forms a ternary complex together with its ligand and associated G proteins^7^. More recently, increasing evidence indicates that APJ may interact with other GPCRs to form heterodimers, which may selectively modulate distinct intracellular signal transduction pathways^2^,^8^,^9^. However, the existence and distribution of APJ homodimers and higher-order oligomers, and their interfaces and potential functions, remains controversial and has been extensively debated.

Here, we explored the characteristics of human APJ using total internal reflection fluorescence microscopy (TIRFM), resonance energy transfer, mass spectrometry (MS), and proximity biotinylation. Our results successfully demonstrated the formation of APJ homodimers and oligomers at the single-molecule level. In cells with <0.3 receptor particles/μm^2^, approximately 60% of APJ molecules existed as homodimers or oligomers. Bioluminescence resonance energy transfer (BRET) and bimolecular fluorescence complementation (BiFC) also confirmed the existence of APJ homodimers and oligomers. Furthermore, our results also demonstrated homodimer and oligomer formation was independent of agonist stimulation, but the density of APJ was more prominent for oligomer. APJ homodimers were present on the cell surface in a dynamic equilibrium with constant formation and dissociation of new receptor complexes. Notably, HIV TAT-fused transmembrane domain (TMD) interference peptides (TMD1, TMD2, TMD3, and TMD4) disrupted APJ homodimerization *in vitro*. Moreover, different quaternary structures of APJ demonstrated novel signaling and functional characteristics, as APJ dimers activated only Gαi3 and Gαq, while distinct cellular responses were observed after agonist stimulation by xCELLigence instruments. Thus, APJ dimers could be a promising target for the development of more selective drugs that have fewer side effects.

## Results

### Visualization and analysis of APJ receptors on the surface of living cells

Prior to exploring the characteristics of APJ, single GFP molecules were analyzed^10^,^11^. To obtain GFP, a pET-21a (+)-GFP plasmid was constructed and transfected into BL21(DE3) cells. GFP was induced for 5 h by addition of IPTG when OD600 reached 0.7. Then, GFP was extracted (Fig. S1) and scattered on a FluoroDish via a glutaraldehyde cross-linking method. Individual GFP proteins were detected and visualized by TIRFM (488 nm) (Fig. S1A). The distribution of the intensity of 321 particles was analyzed and fitted to a Gaussian function. The results indicated a predominant peak, and the average intensity was 308.08 ± 3.38 (mean ± SD) (Fig. S1B).

**Figure 1:**
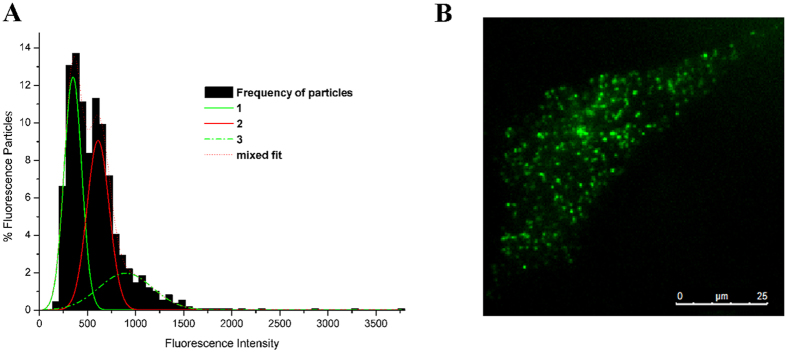
Visualization and quantitative analysis of green fluorescent protein (GFP)-tagged apelin receptor (APJ) on the surface of living cells. (**A**) Distribution of the intensity of APJ-GFP particles (n = 1087). Three peaks were observed for APJ-GFP (green, red and dotted green curves) (1 = monomer, 2 = dimer, 3 = oligomer). (**B**) Total internal reflection fluorescence microscopy (TIRFM) image of Chinese hamster ovary (CHO) cells expressing APJ-GFP scattered on a FluoroDish. Four independent experiments at least were performed and data were listed as a mean.

Next, we applied the methods described in the *SI* to visualize APJ-GFP (the C-terminus of APJ was labeled with green fluorescent protein (GFP)-tags with precise 1:1 APJ/GFP ratio)^12^. Chinese hamster ovary (CHO) cells were transfected with APJ-GFP and observed by TIRFM (488 nm) (Fig. 1). To avoid serial and stochastic fluorophores in the sample and temporally separated molecules that would be spatially indistinguishable, APJ-GFP expression levels were controlled (measured 8 h after transfection to ensure low expression levels [less than one receptor particle/μm^2^]) so that the protein particles were counted and distinguished properly.

The distribution of the intensity of 1087 APJ-GFP particles was analyzed and fitted to a *Gaussian* function (equation (2)). As shown in Fig. 1A, the signal intensities of the individual spots fitted to three Gaussian distributions (a largely predominant peak (green curve), a peak of approximately double intensity (red curve), and a smaller peak of approximately three-fold intensity (green dotted curve). The average intensities were 311.7 ± 12.37, 610.48 ± 5.85, and 896.87 ± 21.86 (mean ± SD), respectively. Given the results from the analysis of a single GFP molecule (Fig. S1), this revealed a mixed distribution of APJ monomers, dimers, and oligomers (1 = monomer, 2 = dimer, 3 = oligomer) (Fig. 1A). With 1760 spots from 11 cells, 40% were identified as monomers, while 36% were dimers, and 24% were oligomers (Four independent experiments were performed and data were listed as a mean). The proportion of monomers, dimers, and higher-order oligomers increased with particle density (<0.13 receptor particles/μm^2^) and reached an equal proportion with >0.13 receptor particles/μm^2^. Notably, monomers and homodimers were present even at very low receptor densities (0.04 receptor particles/μm^2^), while higher-order oligomers were found only at particle densities higher than 0.09, indicating APJ oligomers were more prominent at higher densities (Fig. S2). APJ monomers, dimers, and oligomers were distinguished according to the smallest normalized intensity and using equation (1) (Fig. 1B).

### Formation of APJ homodimers and oligomers is agonist independent

**Figure 2:**
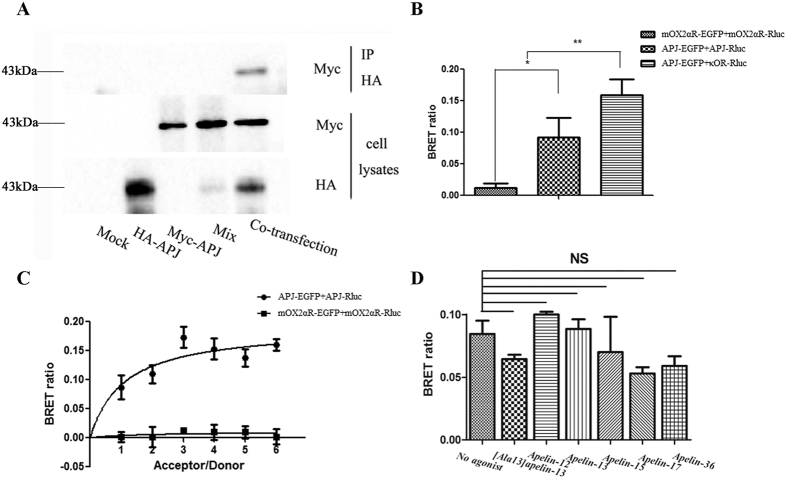
Assessment of apelin receptor (APJ) dimerization using co-immunoprecipitation (Co-IP) and bioluminescence resonance energy transfer (BRET) assays. (**A**) Assessment of APJ homodimerization with Co-IP. Chinese hamster ovary (CHO) cells were either not transfected (mock) or transfected with HA-APJ, Myc-APJ, or both (co-transfection). HA-APJ and Myc-APJ were also mixed (mix). Immunoprecipitated samples were resolved by SDS-PAGE and immunoblotted with an anti-Myc antibody (upper panel). Cell lysates were examined by immunoblotting with either an anti-Myc or anti-HA antibody (lower panels). (**B**) Assessment of APJ homodimerization with BRET^1^. (**C**) BRET saturation curves. (**D**) Assessment of effects of agonist-treatment on dimerization by BRET^1^. For BRET, four independent experiments were performed with triplicate samples and the results were expressed as the mean ± SEM of four experiments (*P < 0.01, **P < 0.05 versus control group; NS, not significant versus unstimulated cells).

We further assessed APJ interactions by co-immunoprecipitation (Co-IP), BRET^1^, and Förster resonance energy transfer (FRET). Co-IP analysis of hemagglutinin (HA)-tagged APJ and Myc-APJ in co-transfected CHO cells revealed that immunoreactive bands against the anti-HA and anti-Myc antibodies were evident only when both receptors were co-expressed (Fig. 2A). BRET studies were performed to confirm APJ homodimerization. In BRET saturation assays, CHO cells co-transfected with APJ-Rluc and APJ-EGFP resulted in a saturation curve, indicating APJ self-interaction. By contrast, the negative controls, mOX2αR-Rluc and mOX2αR- EGFP^13^, induced BRET signals increased with increasing concentrations of mOX2αR-EGFP in a quasi-linear manner (Fig. 2B,C), indicating a non-specific interaction. Furthermore, when cells co-expressing APJ-Rluc and APJ-EGFP (1:1) were treated with an antagonist (^[Ala13]^apelin-13) or agonist (apelin-12, 13, 15, 17, 36), BRET signals were not significantly different to those of unstimulated cells (Fig. 2D), indicating that the formation of APJ homodimers is antagonist and agonist independent.

**Figure 3:**
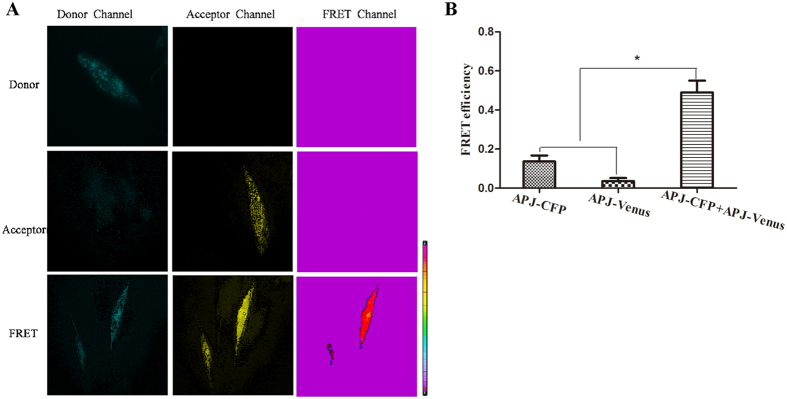
Assessment of apelin receptor (APJ) dimerization with Förster resonance energy transfer sensitized emission (FRET-SE). (**A**) FRET images. Donor group: APJ-CFP only; Acceptor group: APJ-Venus only; FRET group: APJ-CFP and APJ-Venus. (**B**) APJ homodimer FRET efficiency. Four independent experiments were performed with duplicate samples and the results were expressed as the mean ± SEM of four experiments (*P < 0.01 versus control group).

FRET sensitized emission (SE) was also performed to provide further evidence for APJ homodimerization in living cells. Correction factors β and γ showed the highest crosstalk values, whereas α and δ were rather small; α indicated 18% crosstalk for the acceptor channel into the donor channel; β indicated 60% crosstalk for the donor channel into the FRET channel; γ indicated 52% crosstalk for the acceptor channel into the FRET channel; and δ indicated 35% crosstalk for the donor channel into the acceptor channel (Table S1). No FRET signals were detectable in cells transfected with APJ-CFP or APJ-Venus alone. However, a notable FRET signal was detected in CHO cells co-transfected with APJ-CFP and APJ-Venus (Fig. 3A) and FRET efficiency was 52% calculated by equation (3) (Fig. 3B), indicating that APJ exists as a homodimer. Notably, as a result of membrane fluidity, the FRET signal was variable, which was consistent with monomer-dimer-oligomer dynamics on cell membranes (Movie 1).

**Figure 4:**
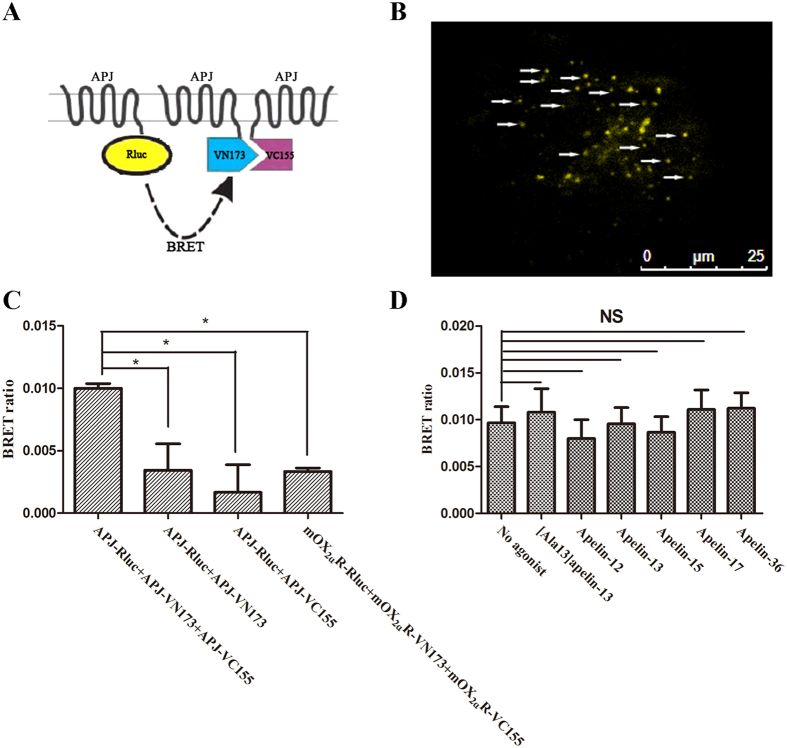
Assessment of apelin receptor (APJ) oligomerization with bioluminescence resonance energy transfer - bimolecular fluorescence complementation (BiFC-BRET). (**A**) Principle of BiFC-BRET. The assay examines the formation of functional Venus by the complementation of protein fragments fused to APJ. Association of APJ enables formation of a bimolecular fluorescent complex. If all three tagged APJ proteins interact, BRET can occur. (**B**) TIRFM detection of APJ dimers (yellow particles) after BiFC. (**C**) Assessment of APJ oligomerization with BiFC-BRET. (**D**) Assessment of agonist-induced oligomerization. Four independent experiments were performed with triplicate samples and the results were expressed as the mean ± SEM of four experiments (*P < 0.01 versus control group; NS, not significant versus unstimulated cells).

Additionally, BiFC-BRET assays were performed to detect APJ oligomerization in living cells. Two non-fluorescent fragments (pCE-BiFC-VN173 and pCE-BiFC-VC155) of Venus fluorescent protein were separately fused to APJ (APJ-VN173 and APJ-VC155) (Fig. 4A). CHO cells were then co-transfected with APJ-Rluc, APJ-VN173, and APJ-VC155 and observed with TIRFM. Detectable Venus fluorescence indicated that BiFC occurred, thereby indicating an interaction between APJ proteins (Fig. 4B). Moreover, a high BRET ratio was detected, which demonstrated the existence of APJ oligomers (Fig. 4C). Furthermore, when cells co-expressing these receptors were treated with an antagonist ([Ala^13^]apelin-13) or an agonist (apelin-12, 13, 15, 17, 36), the BRET ratio was the same as that of unstimulated cells (Fig. 4D), indicating that antagonist and agonist treatment did not induce receptor oligomerization.

### TMD1, 2, 3, and 4 provide APJ dimer interfaces

**Figure 5:**
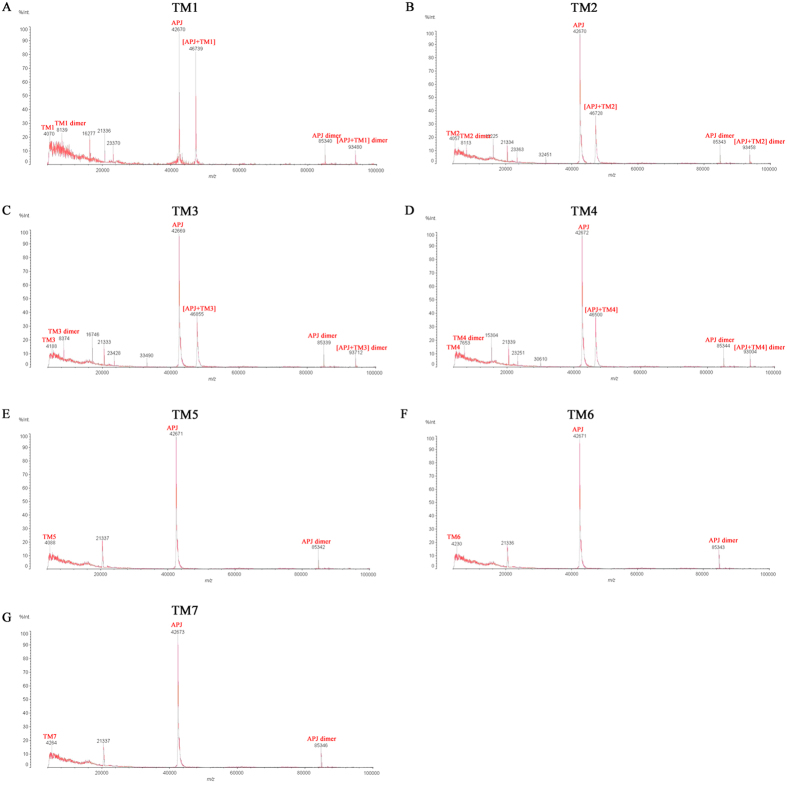
The effects of interference peptides on apelin receptor (APJ) homodimerization as measured by matrix assisted laser desorption tandem time-of-flight (MALDI-TOF) mass spectrometry. (**A**) transmembrane domain 1 (TMD1), TMD1 dimers, TMD1 tetramers, APJ, [APJ + TMD1], APJ dimers, and [APJ + TMD1] dimers were detected (TMD1 MW = 4067.95). (**B**) TMD2, TMD2 dimers, TMD2 tetramers, APJ, [APJ + TMD2], APJ dimers, and [APJ + TMD2] dimers were detected (TMD2 MW = 4054.84). (**C**) TMD3, TMD3 dimers, TMD3 tetramers, APJ, [APJ + TMD3], APJ dimers, and [APJ + TMD3] dimers were detected (TMD3 MW = 4186.01). (**D**) TMD4, TMD4 dimers, TMD4 tetramers, APJ, [APJ + TMD4], APJ dimers, and [APJ + TMD4] dimers were detected (TMD4 MW = 3824.75). (**E**) TMD5, APJ, and APJ dimers were detected (TMD5 MW = 4085.9). (**F**) TMD6, APJ, and APJ dimers were detected (TMD6 MW = 4228.27). (**G**) TMD7, APJ, and APJ dimers were detected (TMD7 MW = 4261.10).

TMDs are important for the formation of head-to-head interfaces in class A GPCR dimers^14^,^15^,^16^,^17^,^18^. To identify dimerization interfaces in the seven TMDs of APJ, the effects of cell-penetrating interference peptides (containing the sequence of the hydrophobic transmembrane helices) on APJ homodimerization were examined by MS. The sequence and molecular weight of each TMD are shown in Table 1 and Fig. S3. As shown in Fig. 5A, TMD1, TMD1 dimers, APJ, [APJ+TMD1], APJ dimers, and [APJ+TMD1] dimers were detected, suggesting that TMD1 is involved in the APJ self-interaction. Similar results were observed with TMD2, 3, and 4 (Fig. 5B,C,D). By contrast, TMD5, 6, and 7 lacked the effect demonstrated by TMD1, 2, 3, and 4 (Fig. 5E,F,G). The results indicate that TMD1, 2, 3, and 4 provide the most notable APJ dimer interfaces, not TMD5, 6 and 7.

**Figure 6:**
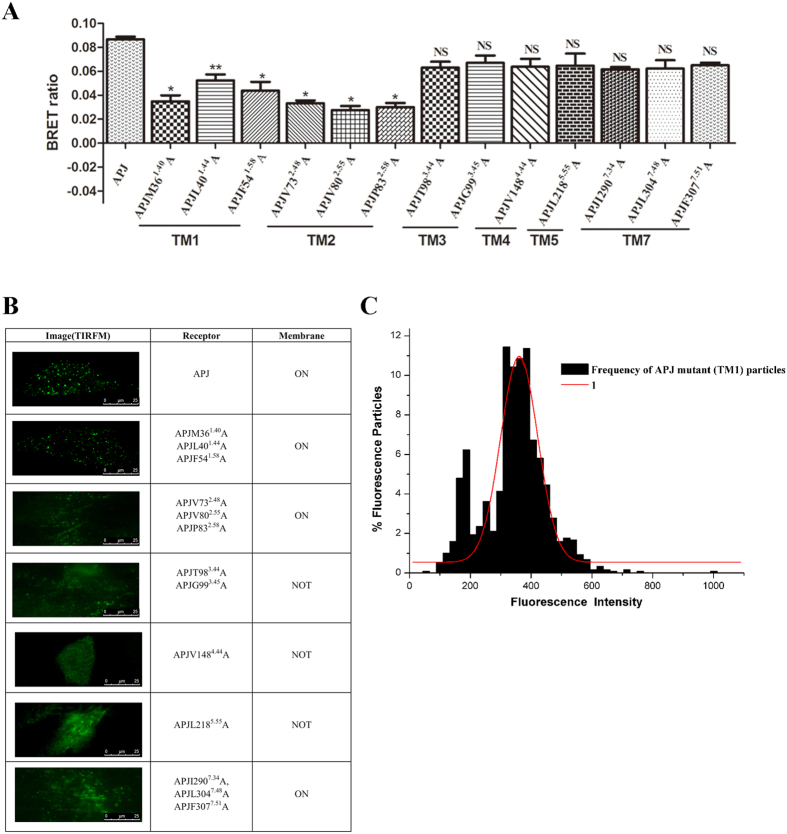
Identification of key amino acids involved in forming apelin receptor (APJ) dimer interfaces by point mutation. (**A**) Detection of a series of APJ mutants by BRET^1^. Four independent experiments were performed with triplicate samples and the results were expressed as the mean ± SEM of four experiments (*P < 0.01, **P < 0.05, NS, not significant versus wt APJ). (**B**) Visualization of a series of APJ mutants by total internal reflection fluorescence microscopy (TIRFM) in living cells. (**C**) Distribution of the intensity of 1189 individual APJ-GFP mutant particles. One peak (red curve) was observed.

**Figure 7:**
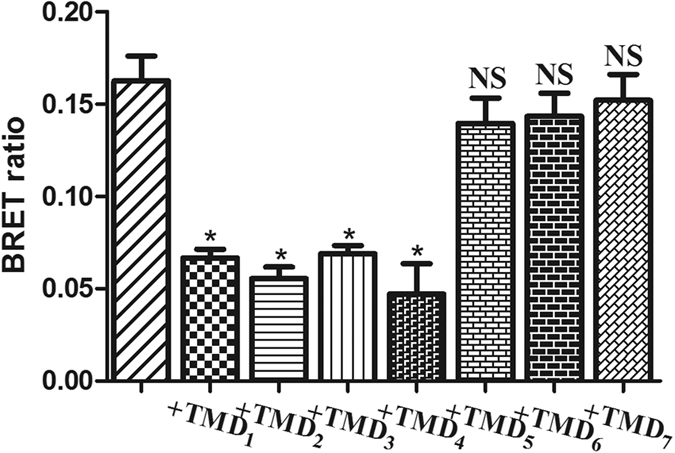
The effect of interference peptides on apelin receptor (APJ) dimers. Chinese hamster ovary (CHO) cells were co-transfected with APJ-Rluc and APJ-EGFP (1:3) and incubated with interference peptides corresponding to transmembrane domain 1 (TMD1), TMD2, TMD3, TMD4, TMD5, TMD6, or TMD7 at 37 °C (4 μM). BRET^1^ was detected as described above. Four independent experiments were performed with triplicate samples and the results were expressed as the mean ± SEM of four experiments (*P < 0.01; NS, not significant versus untreated (with TMD peptides) cells).

To confirm the MS results that revealed the importance of TMD1, 2, 3, and 4 in the formation of APJ dimer interfaces, point mutations were constructed to explore the residues mediating APJ interactions according to previous reports^19^,^20^. Briefly, we generated 13 mutant receptors including “outward-facing” residues APJM36^1.40^A, APJL40^1.44^A, APJV73^2.48^A, APJV148^4.44^A, and APJI290^7.34^A, as well as hydrophobic residues such as APJF54^1.58^A, APJV80^2.55^A, APJP83^2.58^A, APJT98^3.44^A, APJG99^3.45^A, APJL218^5.55^A, APJL304^7.48^A, and APJF307^7.51^A^21^. Plasmids encoding Rluc-tagged mutant APJ receptors and EGFP-tagged wild-type (wt) APJ were transfected into CHO cells, followed by BRET^1^ detection as described above (Fig. 6A). Since the correct membrane location is a prerequisite for BRET, to exclude the possibility of incorrect APJ localization leading to a decreased BRET ratio, all mutants were observed by TIRFM before BRET measurement (Fig. 6B). Only mutants with the correct membrane localization were used for BRET measurements. Notably, the APJM36^1.40^A, APJL40^1.44^A, and APJF54^1.58^A mutants in TMD1, and the APJV73^2.48^A, APJV80^2.55^A, APJP83^2.58^A mutants in TMD2 exhibited significantly lower BRET signals than wt APJ, also highlighting the importance of TMD1 and 2 in the formation of the APJ dimer interface. Notably, the mutations in TMD3 and 4 had little effect on the BRET signals, indicating that these mutations may not induce significant conformational changes in TMD3 and 4, or that TMD3 and 4 may be less important for APJ homodimerization *in vitro* (Fig. 6A). Moreover, the distribution of the intensity of 1189 APJL40^1.44^A-GFP (TMD1) particles was analyzed and fitted to a Gaussian function. Only one peak was observed for APJL40^1.44^A (TMD1) (Fig. 6C), also suggesting that the Leu40^1.44^ (TMD1) mutation disrupted the formation of APJ homodimers. Furthermore, the effects of the TMDs on APJ dimers were explored with BRET^1^. CHO cells co-transfected with APJ-Rluc and APJ-EGFP (1:3) were incubated with TMD1, 2, 3, 4, 5, 6, or 7 peptides at 37 °C (4 μM) and detected by BRET^1^. As shown in Fig. 7, TMD1, 2, 3, and 4 significantly reduced APJ dimer BRET signals (by 55–75%), while TMD5, 6, and 7 had no significant effect. Thus, peptides corresponding to TMD1, 2, 3, and 4 can disrupt the formation of APJ homodimers, further suggesting the involvement of these TMDs in the APJ homodimer interface (Fig. 7).

### Measurement of APJ receptor homodimer dynamics on cell membranes

**Figure 8:**
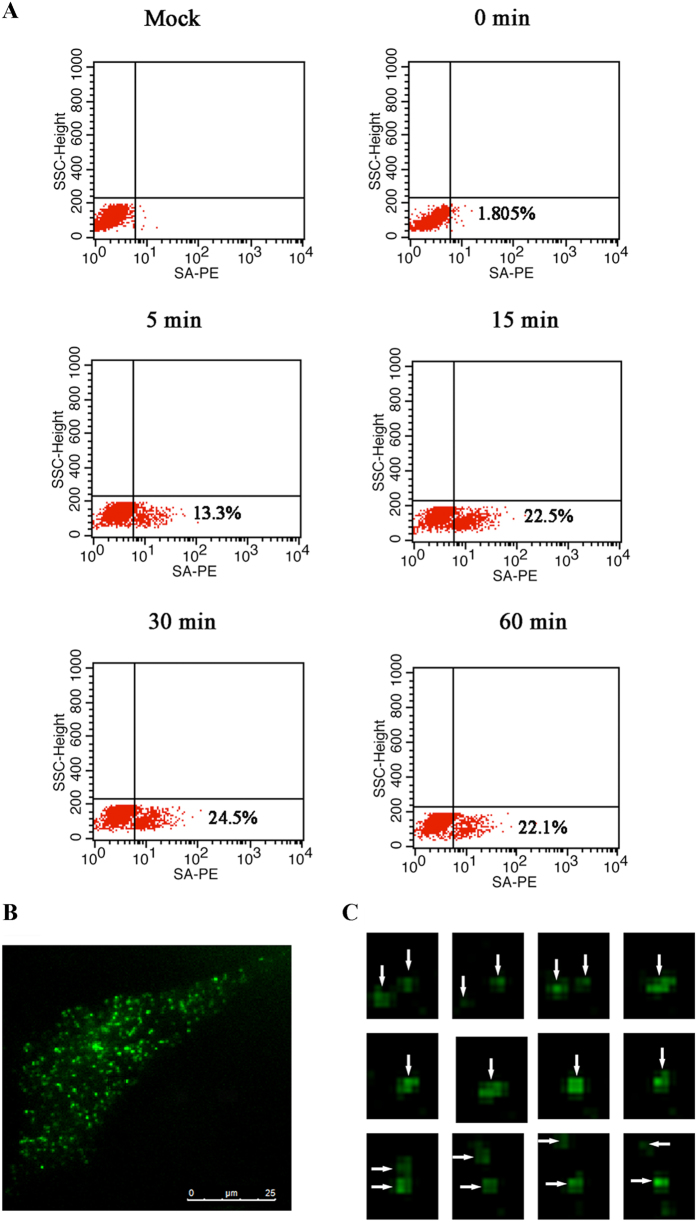
Measurement of apelin receptor (APJ) homodimer dynamics. (**A**) Detection of biotinylated APJ with streptavidin-phycoerythrin (SA-PE) and flow cytometry. Biotinylation increased linearly within ~15 min and reached saturation after 15 minutes. The results were analyzed using FlowJo software. (**B**) Single-molecule co-tracking of APJ dimers by total internal reflection fluorescence microscopy (TIRFM). APJ proteins were initially monomers, formed a dimer for ~900 ms, and then rapidly dissociated into monomers. Four independent experiments were performed and data were listed as a mean.

Proximity biotinylation was also used to examine APJ homodimer dynamics. Since biotin and streptavidin form a tight interaction with a slow off-rate (of days), biotinylated APJ would remain biotinylated even if the dimers dissociated. Here, we detected biotinylation using immunofluorescence imaging and found that acceptor peptide (AP)-APJ can be biotinylated, indicating the formation of APJ dimers (Fig. S4). Subsequently, we observed that the biotinylation increased linearly within ~15 min and reached saturation after 15 minutes, which likely reflects the continuous association and dissociation of APJ in a monomer-dimer dynamic equilibrium (Fig. 8A).

Proteins, including APJ, present on the membrane of living cells are typically dynamic (Movie 2); therefore, APJ could interact by coincidence and be falsely identified as an interaction partner, which represents one of the pitfalls of single-molecule co-tracking. Thus, we detected single-molecule co-tracking of APJ dimers by TIRFM and the co-tracking method was restrained to particle densities of <1 particle/μm^2^. The images indicated APJ exists in a dimeric form with short dimer lifetimes (Fig. 8B).

### Novel G protein exchange induced by APJ dimers

**Figure 9:**
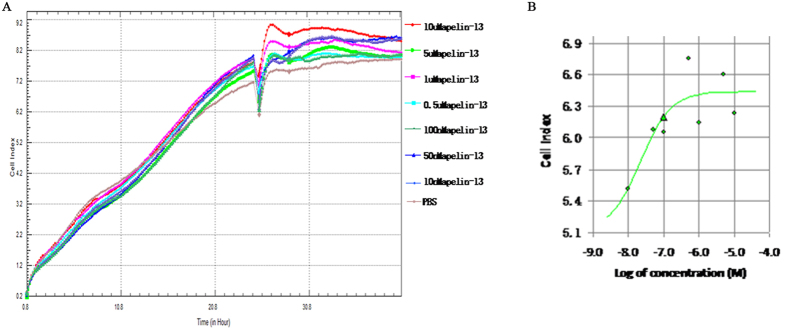
Concentration for 50% of maximal effect (EC50) with xCELLigence Real-Time Cell Analyzer (RTCA). (**A**) The different concentrations of apelin-13 (0 uM, 0.01 uM, 0.05 uM, 0.1 uM, 0.5 uM, 1 uM, 5 uM, 10 uM, 100 uM) were added. (**B**) The results were analyzed by RTCA Software 2.0. All curves were plotted as the average of quadruplicate treatments, with error bars shown as SEM.

**Figure 10:**
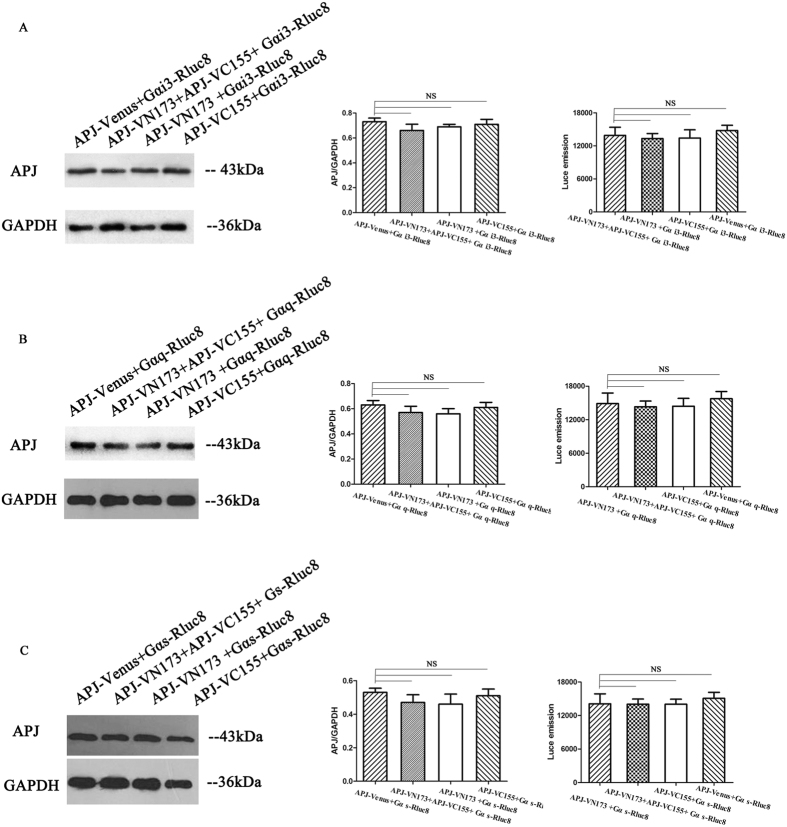
Expression levels of donor and acceptor were measured before BiFC-BRET experiment. (**A**) Gαi3-Rluc8, APJ-VN173, and APJ-VC155; (**B**) Gαs-Rluc8, APJ-VN173, and APJ-VC155. The expression levels of donor measured with BRET and expression levels of acceptor were measured with western blot. Four independent experiments were performed and the results were expressed as the mean ± SEM of four experiments (NS, not significant versus control group).

**Figure 11:**
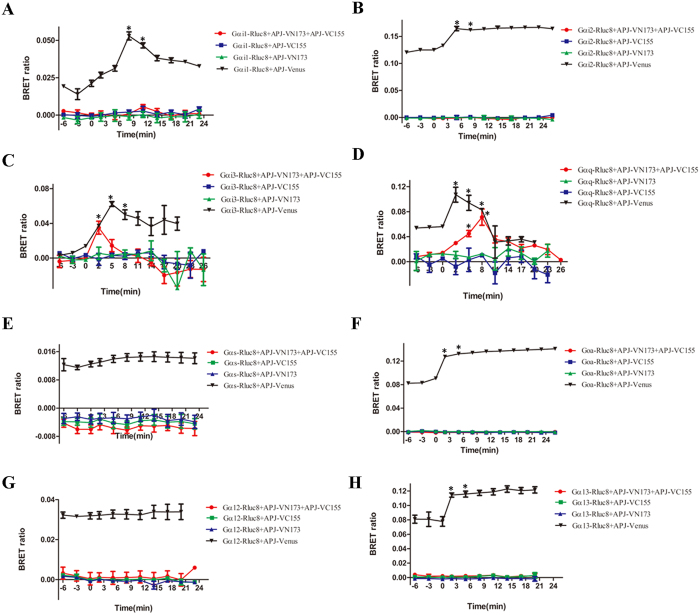
Identification of novel G-protein coupling to apelin receptor (APJ) homodimers by BiFC-BRET. Kinetics of ligand-induced BRET between Gα-Rluc8 (8 subtypes), APJ-VN173, and APJ-VC155. BRET was measured after addition of 1 μM apelin-13 for 30 min. (**A**) Gαi1-Rluc8, APJ-VN173, and APJ-VC155; (**B**) Gαi2-Rluc8, APJ-VN173, and APJ-VC155; (**C**) Gαi3-Rluc8, APJ-VN173, and APJ-VC155; (**D**) Gαq-Rluc8, APJ-VN173, and APJ-VC155; (**E**) Gαs-Rluc8, APJ-VN173, and APJ-VC155; (**F**) Gαo-Rluc8, APJ-VN173, and APJ-VC155; (**G**) Gα12-Rluc8, APJ-VN173, and APJ-VC155; H. Gα13-Rluc8, APJ-VN173, and APJ-VC155. Four independent experiments at least were performed with triplicate samples and the results were expressed as the mean ± SEM (*P < 0.01 versus unstimulated cells).

GPCR dimers can exhibit novel signaling, different from that of their monomers. To explore the novel signaling of APJ dimers, we examined the signaling of APJ dimers using BiFC-BRET. First, we detected concentration for 50% of maximal effect (EC50) with xCELLigence Real-Time Cell Analyzer (RTCA). The different concentrations of apelin-13 (0 uM, 0.01 uM, 0.05 uM, 0.1 uM, 0.5 uM, 1 uM, 5 uM, 10 uM, 100 uM) were added (Fig. 9A). The results were analyzed by RTCA Software 2.0, showing EC50 of APJ was 2.25 × 10^−8^ M and concentration for maximal effect was 10^−6^ M (Fig. 9B). For G protein dependent signaling, Gα-Rluc8, APJ-VN173, and APJ-VC155 were transfected into CHO cells and BRET signals measured after treatment with 1 μM apelin-13. Cells expressing Gα-Rluc8 and APJ-Venus served as a positive control, and two negative controls (Gα-Rluc8 and APJ-VN173, Gα-Rluc8 and APJ-VC155A) were also introduced. These tags have been characterized and do not significantly alter the function of the wildtype APJ^22^ (Fig. S5). To express a similar level, the mount of plasmid in positive controls and negative controls were transfected, same as that in experiment group. In addition, we have measured the expression levels of donor^23^ and acceptor before experiment (Fig. 10). With the positive controls, the increase in BRET signal was rapid, reaching a maximum ~5 min after apelin-13 addition for Gαi1, Gαi2, Gαi3, Gαq, and Gαo, while with cells expressing APJ tagged with VN173 and VC155, an increase in BRET was observed only for Gαi3 and Gαq. No changes in BRET were observed with the negative controls (Fig. 11). The results indicate that APJ monomers activate Gαi1, Gαi2, Gαi3, Gαq, Gα13, and Gαo, but not Gαs and Gα12, while APJ dimers activate only Gαi3 and Gαq, but not Gαi1, Gαi2, Gαs, Gα12, Gα13, and Gαo.

### Disruption of APJ dimerization results in distinct cell responses after apelin-13 stimulation

**Figure 12:**
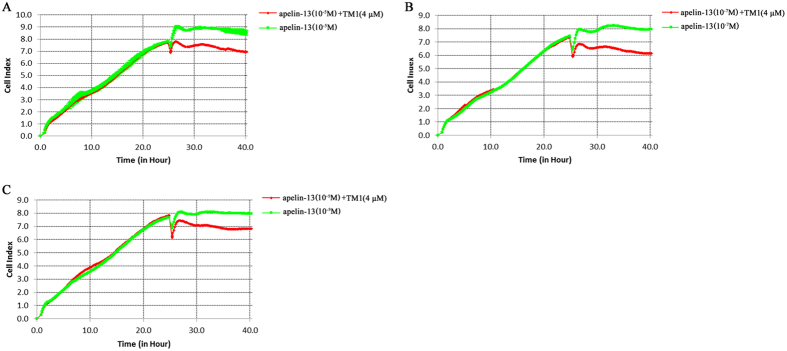
Effect of interference peptides on apelin-13-induced cellular responses. Growth curves of human umbilical vein endothelial cells (HUVECs) treated with 10 μM (**A**), 0.1 μM (**B**), and 1 nM (**C**) apelin-13 (green line). Peptide-treated cells were treated with transmembrane domain 1 (TMD1) (4 μM) and apelin-13 (red line). All curves were plotted as the average of quadruplicate treatments, with error bars shown as SEM.

Our previous study demonstrated that APJ monomers and APJ-bradykinin 1 receptor (B1R) heterodimers show different effects on the regulation of eNOS phosphorylation in human umbilical vein endothelial cells (HUVECs)^24^ eNOS is involved in the regulation of endothelial cell proliferation and/or migration. Here, we also measured the effects of interference peptides on apelin-13-induced cellular responses in HUVECs, as measured with an xCELLigence Real-Time Cell Analyzer. As shown in Fig. 12, apelin-13 stimulation elicited a further increase in the HUVEC cell index (CI); however, co-stimulation with interference peptides corresponding to TMD1, which was demonstrated to disrupt APJ homodimers, led to a decreasing trend in the CI compared to that before treatment, suggesting disruption of APJ homodimers induced distinct cell responses after agonist stimulation.

## Discussion

GPCR dimerization and oligomerization *in vivo* and *in vitro* has been demonstrated by a range of techniques including co-IP, resonance energy transfer, proximity ligation assays, and protein fragment complementation assays^25^,^26^. Here, we examined APJ homodimerization and oligomerization with BRET, FRET, MS, TIRFM, and traditional Co-IP. Although previous studies demonstrated the existence of APJ heterodimerization with other GPCRs *in vitro*, no study to date has revealed the characteristics of human APJ homodimers and oligomers. Here, analyses of BRET, BiFC, and traditional Co-IP data provide solid evidence for the formation of functional APJ homodimers and oligomers. Furthermore, using single-molecule fluorescence imaging methods (TIRFM), we revealed monomer-to-dimer interconversion of APJ molecules on the cell membrane. TIRFM provides advantages for monitoring membrane receptors within a thin layer 100 nm from the coverslip and improves the detection of single fluorescent molecules on the membrane^27^,^28^,^29^. Therefore, our observations likely reflect the dynamics of APJ on the cell membrane.

We also measured the dynamics of the quaternary organization of APJ by observing GFP-labeled APJ as individual, mobile, fluorescent spots that were evenly distributed on the cell surface. In cells with less than 0.3 receptor particles/μm^2^, the proportion of APJ monomers, dimers, and oligomers was ~40%, ~36%, and ~24%, respectively. Agonist stimulation further increased the density of APJ oligomer, which has also been reported by several other recent studies in which agonist stimulation promoted GPCR dimerization^30^. Notably, even in the absence of extracellular stimulation, many receptors form dimers, including epidermal growth factor receptor^31^ and various GPCRs^32^,^33^,^34^. Moreover, our observations also indicate that APJ receptors on the cell surface are in a dynamic equilibrium with constant formation and dissociation of new receptor complexes, which was also demonstrated previously^30^,^35^. Recently, using single-particle tracking experiments in cells expressing GFP-tagged adenosine A1 and mCherry-tagged A2A receptors, a preponderance of cell surface 2:2 receptor heteromers (dimer of dimers) that coupled with two G proteins was identified, suggesting the quaternary structure of the GPCR may be involved in the molecular intricacies of GPCR function^12^.

Additionally, we examined the interacting interfaces of APJ dimers using interference peptides and MS. The results demonstrated that peptides encoding TMD1, TMD2, TMD3, and TMD4 can bind to APJ, suggesting these TMDs are involved in interface formation. To the best of our knowledge, this is the first time that MS and interference peptides were utilized to demonstrate the GPCR dimer and examine dimer interfaces simultaneously. Given the high sensitivity and accuracy of the mass analyzer, this method could provide extremely useful information for understanding GPCR structure and conformation changes during the formation of dimers. Furthermore, point mutations in TMD1 and TMD2 also disrupted the APJ dimers, which is consistent with previous findings that TMD1 and TMD2 are important for GPCR dimerization^36^,^37^,^38^. It is most likely that two of these are the dimer and two drive the tetramer interaction, which needs further investigation. Notably, these finding also highlight the therapeutic potential of interference peptides, as they can disrupt dimerization and influence receptor function. Indeed, Bouvier *et al*. showed that a peptide derived from TMD6 of β2AR could disrupt the dimer and decrease receptor function^39^. Moreover, BRET also revealed that peptides corresponding to TMD1, 2, 3, and 4 can effectively disrupt the formation of APJ homodimers, which highlights the potential of these peptide for targeting distinct dimer-specific physiological effects.

Recent studies demonstrate that GPCR dimerization is of physiological relevance. In general, receptor dimerization or oligomerization is important in many signaling pathways, often as the first step for inducing intracellular signals upon ligand binding^40^,^41^,^42^. For example, orexin receptor–corticotropin-releasing factor receptor heteromers in the ventral tegmental area serve as targets for cocaine and promote long-term disruption of orexin-A–CRF negative crosstalk^37^. Therefore, GPCR dimer-monomer interconversion may be important for precise control of signal transduction. Here, APJ dimers induced distinct signaling and functional characteristics from those of monomers; APJ dimers activated only Gαi3 and Gαq. These novel characteristics will help develop more selective drugs that have fewer side effects. Recent studies demonstrate that monovalent drugs specific for GPCR heterodimers^43^ and bivalent ligands^44^,^45^ are powerful tools for evaluating activation of signaling pathways and cellular responses elicited by GPCR dimerization and oligomerization. Further studies are needed to generate specific agonists for APJ dimers/oligomers to investigate the novel G-protein binding characteristic of APJ dimers.

Our results also revealed distinct cellular responses profiles in interference peptide-treated versus non-interference peptide-treated HUVECs after apelin-13 stimulation. Recent studies used the xCELLigence system to monitor activation of endogenous GPCRs. For example, administration of UK14,304 (0.15 to 333 nM) to CHO-K1 cells expressing human α-adrenergic 2 A receptors (Gαi coupled) induces a dose-dependent increase in CI. Stimulating serum-deprived CHO-K1 cells with calcitonin (5 pM to 5 μM) also resulted in a transient agonist-induced increase in CI lasting up to a few hours. Here, we also observed a transient increase in HUVEC CI after apelin-13 stimulation for 2–3 h; this observation is consistent with previous findings. Furthermore, interference peptides abolished these apelin-13-induced changes, suggesting dimerization may be involved in APJ-mediated morphological or signaling alterations in HUVECs.

Taken together, this study advances our knowledge of the structure and function of APJ; however, much needs to be learned about the neuroanatomy, pharmacology, and signaling of APJ oligomers both *in vitro* and *in vivo* before new therapeutic drugs that affect their function can be developed.

## Methods

### Materials

Chinese hamster ovary cells and human umbilical vein endothelial cells were obtained from American Type Culture Collection (ATCC). All restriction enzymes were from NewEngland BioLabs. [Ala13] apelin-13, apelin-12, 13, 15, 17, 36 were purchased from Phoenix Pharmaceuticals. Lipofectamine 2000, streptavidin-phycoerythrin (SA-PE) and Opti-MEM I were obtained from Invitrogen Life Technologies. Isopropyl-beta-D-thiogalactopyranoside (IPTG) and Coelenterazine h were obtained from Promega. HEPES-buffered phenol red−free medium, Dulbecco’s modified eagle medium and Incomplete RPMI-1640 culture medium were purchased from Gibco. Anti-HA-agarose was obtained from Pierce Chemical Co. Polyclonal horseradish peroxidase-conjugated goat anti-rabbit immuno-globulins/HRP was obtained from Zhong Shan Gold Bridge Biology Corporation (China). Anti-Myc antibody and anti-HA antibody were purchased from Cell Signaling Technology

### Plasmid construction

APJ-VN173 and APJ-VC155 were generated by fusing either the N-terminal fragment of Venus (Venus N: amino acids 1–172) or the C-terminal fragment of Venus (Venus C: amino acids 156–239) to the C-terminus of the wild-type (wt) full-length apelin receptor. Gαi1-Rluc8, Gαi2-Rluc8, Gαi3-Rluc8, Gαq-Rluc8, Gαoa-Rluc8, and Gαs-Rluc8, pET-21a(+)-GFP, FLAG-BirA-APJ, and HA-AP-APJ were constructed as described previously^9^,^46^.

### Cell culture and cDNA transfection

CHO cells were cultured in Dulbecco’s modified Eagle’s medium (DMEM) supplemented with 10% (v/v) fetal bovine serum (FBS; Invitrogen, Life Technologies) at 37 °C/5% CO_2_. HUVECs were cultured in RPMI-1640 medium supplemented with 10% (v/v) FBS at 37 °C/5% CO_2_. Transient transfections were performed with Lipofectamine 2000 M (Invitrogen, Life Technologies) according to the manufacturer’s protocol. Additionally, to ensure low cell expression levels, cells were analyzed 8–12 h after transfection.

### Co-immunoprecipitation

CHO cells were co-transfected with HA-APJ and Myc-APJ or the vector control. 48 h later, cells were lysed. After centrifugation at 4 °C for 15 min at 16,000 *g*, whole cell lysates were incubated with an anti-HA antibody and Protein G-Sepharose beads for 4 h with gentle rotation at 4 °C. The beads were washed four times with cell lysis buffer and precipitates were eluted with 4 × SDS-PAGE sample buffer and analyzed by Western blotting for anti-Myc immunoreactivity. To monitor protein expression levels, cell lysates of each sample were used for Western blots.

### Western blotting

Transfected cells were lysed in lysis buffer. Cell lysates was separated by 10% SDS-PAGE followed by transfer to PVDF membranes. Proteins of interest were probed with primary and secondary antibodies as described above. Enhanced chemiluminescence (ECL) kits were used to visualize protein bands. Films were scanned and bands analyzed using a ChemiDoc MP Imaging System (Bio-Rad).

### Total internal reflection fluorescence microscopy (TIRFM)

A commercial TIRF system (Leica Microsystems) equipped with a 100 × 1.47 NA oil-immersion objective, electron multiplying charge-coupled device camera, and a heatable mounting frame (M-H) was used Illumination was restricted within a single focal plane, with relatively short exposure times (three frames per second), and only 8% laser power for imaging events at the cell surface, thereby avoiding photobleaching during image acquisition. The penetration depth of the evanescent field was ~150 nm. Additionally, the following factors were taken into consideration:

### *Gaussian* fitting

In our experiments, only CHO cells with <0.3 receptor particles/μm^2^ were analyzed. The single-particle tracking algorithm used to identify and track individual APJ receptors has been described previously. APJ monomers, dimers, and oligomers were distinguished according to the smallest normalized intensity and using equation (1):


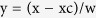


where y is the normalized intensity, w and xc are the standard deviation and the mean, respectively. Receptor particle intensities were analyzed by *Gaussian* fitting (see equation (2)). Briefly, the *Gaussian* fitting was performed with Origin 8.0 software and the following equation (2):


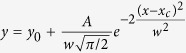


where y is the frequency of the particles and w and xc are the standard deviation and mean, respectively.

### BRET measurement

To monitor constitutive APJ interactions, CHO cells were transfected with APJ-Rluc and APJ-EGFP plasmids at a ratio of 1:1, 1:2, 1:3, 1:4, 1:5, and 1:6 to express a constant amount of donor-labeled protein with increasing amounts of acceptor-labeled protein. At 24 h post-transfection, cells were trypsinized and plated on a 96 well microplate for 24 h in HEPES-buffered phenol red-free medium (Invitrogen, Life Technologies). Coelenterazine h (5 μM; Promega) was added for BRET measurements using a Tristar LB941 plate reader (Berthold) with an Rluc filter (400–475 nm) and a EGFP filter (500–550 nm). To monitor the induced interactions of APJ, BRET signals were measured as described above, with slight modifications. Briefly, cells were transiently transfected with APJ-Rluc and/or APJ-EGFP and stimulated with an antagonist ([Ala^13^]apelin-13) or agonist (apelin-12, 13, 15, 17, 36). To measure the effects of interference peptides on APJ dimers, CHO cells were co-transfected with APJ-Rluc and APJ-EGFP (1:3) and incubated with interference peptides corresponding to TMD1, TMD2, TMD3, TMD4, TMD5, TMD6, and TMD7 at 37 °C (4 μM), and BRET^1^ detected as described above.

### FRET SE

The donor plasmid APJ-CFP and receptor plasmid APJ-Venus were co-transfected into CHO cells as the FRET channel. In addition, the donor and acceptor channel are used to eliminate crosstalk into the FRET channel. FRET sample preparations must therefore include references of the donor in the absence of the acceptor (donor only control) and acceptor in the absence of the donor (acceptor only control). APJ-CFP and APJ-Venus were transfected into CHO cells as donor and acceptor channels, respectively, to obtain calibration coefficients to correct for excitation and emission crosstalk. After 12–24 h, FRET signals were detected with a FRET Kit for a Leica AM TIRF MC system (Leica Microsystems).

### Calculation of FRET efficiency

EA is the apparent FRET efficiency. A, B, C correspond to the intensities of the three signals (donor, FRET, acceptor) and α, β, γ, and δ are the calibration factors generated by the acceptor only and donor only references (equation (3)):


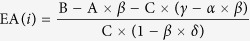


### Design and synthesis of TMD peptides

Peptides derived from human APJ TMDs were custom synthesized, and their primary sequences are shown in Table 1. The identity of the TMD peptide sequences was analyzed with liquid chromatography (LC)-MS. HIV TAT (YGRKKRRQRRR) was fused to the N-terminus of even-numbered TMDs and to the C-terminus of odd-numbered TMDs to obtain the correct orientation for the inserted peptide, because HIV TAT binds to phosphatidylinositol-(4,5)-bisphosphate found on the inner surface of the membrane^47^.

After custom synthesis, the identity of the TMD peptide sequences was analyzed using a LC-MS system (Shimadzu2020 and Water1010). The molecular weight of TMD1, 2, 3, 4, 5, 6, and 7 was 4067.95, 4054.84, 4186.01, 3824.75, 4085.9, 4228.27, and 4261.1 Da, respectively. As shown in Fig. S3, all TMDs were synthesized correctly.

### Mass spectrometry

MS was performed to identity APJ dimer interfaces in samples treated with TMD peptides. Cells were transfected with APJ and, 48 h later, treated with or without the indicated HIV TAT-TMD fused peptides (4 μM) for 60 min at 37 °C. Extracted proteins were immunoprecipitated using an anti-APJ antibody. Protein A/G PLUS-agarose beads were incubated with proteins for 2 h and washed four times with lysis buffer. The APJ complex was eluted from beads as described previously^48^. Then, the endogenous APJ complex was analyzed with an AXIMA matrix-assisted laser desorption/ionization time-of-flight (MALDI-TOF) system (Shimazdu) in linear positive mode. The mass range was 4–10 kDa and the matrix was Sinapic Acid (SA), 10 mg/ml (50% acetonitrile (ACN), 50% H_2_O, 0.1% Sodium trifluoroacetate (TFA)). The results were calibrated with a Shimadzu MALDI calibration kit (Laser BioLabs).

### Proximity biotinylation

*Escherichia coli* biotin ligase (BirA) and an AP were fused to APJ. In the presence of biotin, BirA can site-specifically biotinylate AP. Cells transfected with FLAG-BirA-APJ and HA-AP-APJ were incubated for a designated time (5, 10, 15, and 30 min) with biotinylation media (DMEM, 100 mM biotin, 5 mM MgCl_2_, and 1 mM ATP) at 37 °C. Cells were then rinsed with PBS, detached with pancreatin, pelleted, and resuspended in 1% bovine serum albumin (BSA)/PBS for 15 min. Next, cells were labeled with streptavidin-phycoerythrin (SA-PE) in 1% BSA/PBS. Flow cytometry was performed with a BD FACSCalibur flow cytometer (BD Biosciences). PE was excited by 488 nm laser and emission examined using a 575/24 filter.

### Real-time cell analyzer

An xCELLigence Real-Time Cell Analyzer (RTCA) DP system (ACEA Biosciences) was used to measure the effects of interference peptides on the apelin-13-induced cellular responses as reported via a cell index. A background step (step 1) was performed before other experimental steps. The background step was performed with each well of the E-Plate 16 containing only culture media (100 μl/well) for 30 min at 37 °C, prior to the addition of 10000 HUVECs per well (100 μl/well). Different concentrations of apelin-13 (1 nM, 0.1 μM, and 10 μM) with or without TMD1 (4 μM) were then added during step 3. The schedule settings were as follows (RTCA Software 2.0):


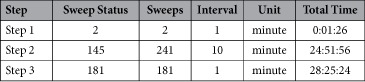


### Statistical analysis

Results were analyzed using GraphPad Prism 5.0 and Origin 8.0 software, and are shown as the mean ± SEM. Student’s paired t-test was used to assess the statistical significance of differences. One-way ANOVA was adopted for multiple group comparisons. P < 0.05 was considered statistically significant.

## Additional Information

**How to cite this article**: Cai, X. *et al*. Apelin receptor homodimer-oligomers revealed by single-molecule imaging and novel G protein-dependent signaling. *Sci. Rep.* **7**, 40335; doi: 10.1038/srep40335 (2017).

**Publisher's note:** Springer Nature remains neutral with regard to jurisdictional claims in published maps and institutional affiliations.

## Supplementary Material

Supplementary Movie 1

Supplementary Movie 2

Supplementary Information

## Figures and Tables

**Table 1:** Amino acid sequences of synthetic peptides derived from the transmembrane domains of APJ.

TMD peptides	Sequence	Molecular weight (Da)
**TMD1**	PAIYMLVFLLGTTGNGLVLWTVF**YGRKKRRQRRR**	4067.95
**TMD3**	IFVNMYASVFCLTGLSFDRYLAI**YGRKKRRQRRR**	4186.01
**TMD5**	VSSTTVGFVVPFTIMLTCYFFIA**YGRKKRRQRRR**	4085.9
**TMD7**	LMNIFPYCTCISYVNSCLNPFLY**YGRKKRRQRRR**	4261.10
**TMD2**	**YGRKKRRQRRR**FIASLAVADLTFVVTLPLWATYT	4054.84
**TMD4**	**YGRKKRRQRRR**VSGAVATAVLWVLAALLAMPVMV	3824.75
**TMD6**	**YGRKKRRQRRR**RLLSIIVVLVVTFALCWMPYHLV	4228.27
