# Supplementary material for: Apelin receptor homodimer-oligomers revealed by single-molecule imaging and novel G protein-dependent signaling
Source: Sci Rep. 2017 Jan 16;7:40335. doi: 10.1038/srep40335 (PMC5238433; doi:10.1038/srep40335)
Supplement: Supplementary Information [file srep40335-s3.pdf]

Apelin receptor homodimer-oligomers revealed by single-molecule imaging and novel G protein-dependent signaling

Xin Cai[a,b], Bo Bai[*b], Rumin Zhang[b], Chunmei Wang[b], Jing Chen[*b,c]

[a]Department of Physiology, School of Medicine, Shandong University, Jinan, Shandong, 250012 P.R. China; [b]Neurobiology Institute, Jining Medical University, Jining, Shandong, 272067 P.R. China; and [c]Division of Biomedical Sciences, Warwick Medical School, University of Warwick, Coventry, CV4 7AL, UK.

Supplementary Information

Fig. S1. Distribution of the intensity of individual green fluorescent protein (GFP) particles. A. Total internal reflection fluorescence microscopy (TIRFM) image of individual GFP particles (green spots) scattered on a FluoroDish. B. Distribution of the intensity of GFP particles (n = 321). One largely predominant peak was observed (red curve). Four independent experiments at least were performed and data were listed as a mean.

Fig. S2. Visualization and quantitative analysis of green fluorescent protein (GFP)-tagged Apelin receptor (APJ) on the surface of living cells at different densities. The proportion of APJ monomers, dimers, and higher-order oligomers in different particle densities analyzed by LAS AF and Origin 8.0 software (1 = monomer, 2 = dimer, 3 = oligomer). Four independent experiments at least were performed and the results were expressed as the mean ± SEM.

Fig. S3. Identification of transmembrane domain (TMD) peptide sequences by liquid chromatography mass spectrometry (LC-MS). A. Identity of TMD1 (Water1010); B. Identity of TMD2 (Shimadzu); C. Identity of TMD3 (Water1010); D. Identity of TMD4 (Shimadzu); E. Identity of TMD5 (Water1010); F. Identity of TMD6 (Water1010); G. Identity of TMD7 (Water1010).

Fig. S4. Detection of apelin receptor (APJ) dimers by proximity biotinylation. Immunofluorescence imaging of streptavidin-phycoerythrin (SA-PE) (red) in Chinese hamster ovary (CHO) cells expressing BirA-APJ and AP-APJ after treatment with biotin for 5 minutes (60× oil-immersion objective). Nuclei were stained with DAPI

(blue).

Fig. S5. Dose-response curve of cAMP accumulation for wild-type (wt) APJ, APJ-Venus, APJ-VN173 and APJ-VC155. CHO cells expressing wtAPJ, APJ-Venus, APJ-VN173 and APJ-VC155 were incubated in 3-isobutyl-1-methylxanthine with (0.1–10,000nM) apelin-13 for 20 min. Intracellular cAMP level was measured with the absorbance-based cAMP ELISA kits. Each independent experiment was performed with triplicate samples. Five independent experiments were performed with triplicate samples and the results were expressed as the mean± SEM of five experiments.

Table S1. Detection of fluorescent resonance energy transfer sensitized emission (FRET-SE) correction factors. α indicated crosstalk for the acceptor channel into the donor channel; β indicated crosstalk for the donor channel into the FRET channel; γ indicated crosstalk for the acceptor channel into the FRET channel; and δ indicated crosstalk for the donor channel into the acceptor channel. Four independent experiments at least were performed and data were listed as a mean.

Movie S1. Visualization of apelin receptor (APJ) dimerization with Förster resonance energy transfer sensitized emission (FRET-SE). The membrane fluidity resulted in the FRET signal was variable**.** Images were collected at three frames per second. To reduce file size, the movie displays only one frame every 5 seconds.

Movie S2. Measurement of apelin receptor (APJ) homodimer dynamics with total internal reflection fluorescence microscopy (TIRFM). GFP-tagged APJ present on the membrane of living cells is monitored by TIRFM. The movie represents the detection and tracking of individual GFP tagged APJ on the surface of living cells. Images were collected at three frames per second.

Figure S1

A

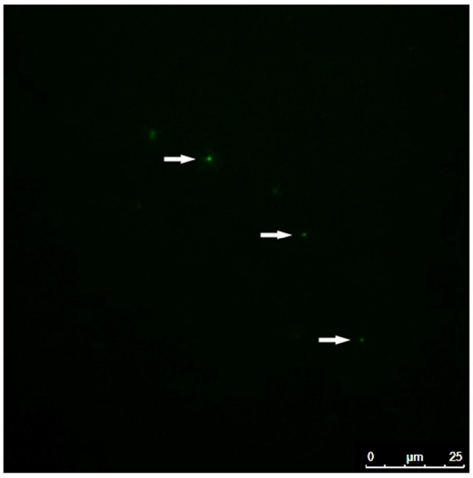
0 µm 25

B

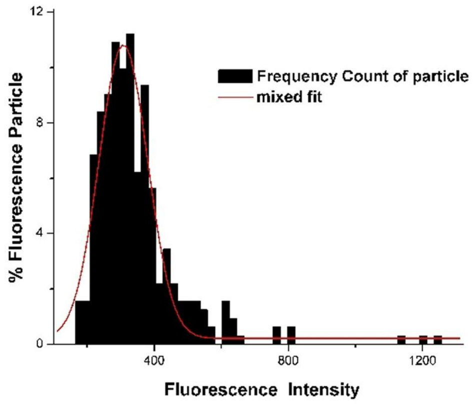
Frequency Count of particle
mixed fit
% Fluorescence Particle
Fluorescence Intensity
12
8
4
0
400
800
1200

Figure S2

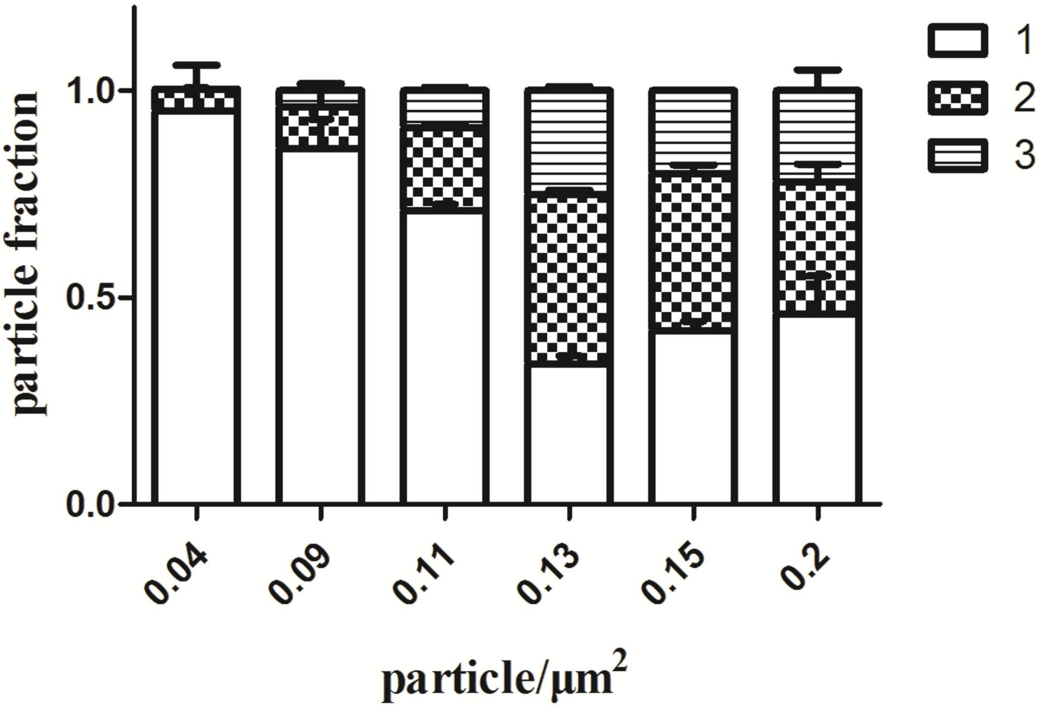

particle fraction
1.0
0.5
0.0
0.04
0.09
0.11
0.13
0.15
0.2
particle/µm²
1
2
3

Figure S3

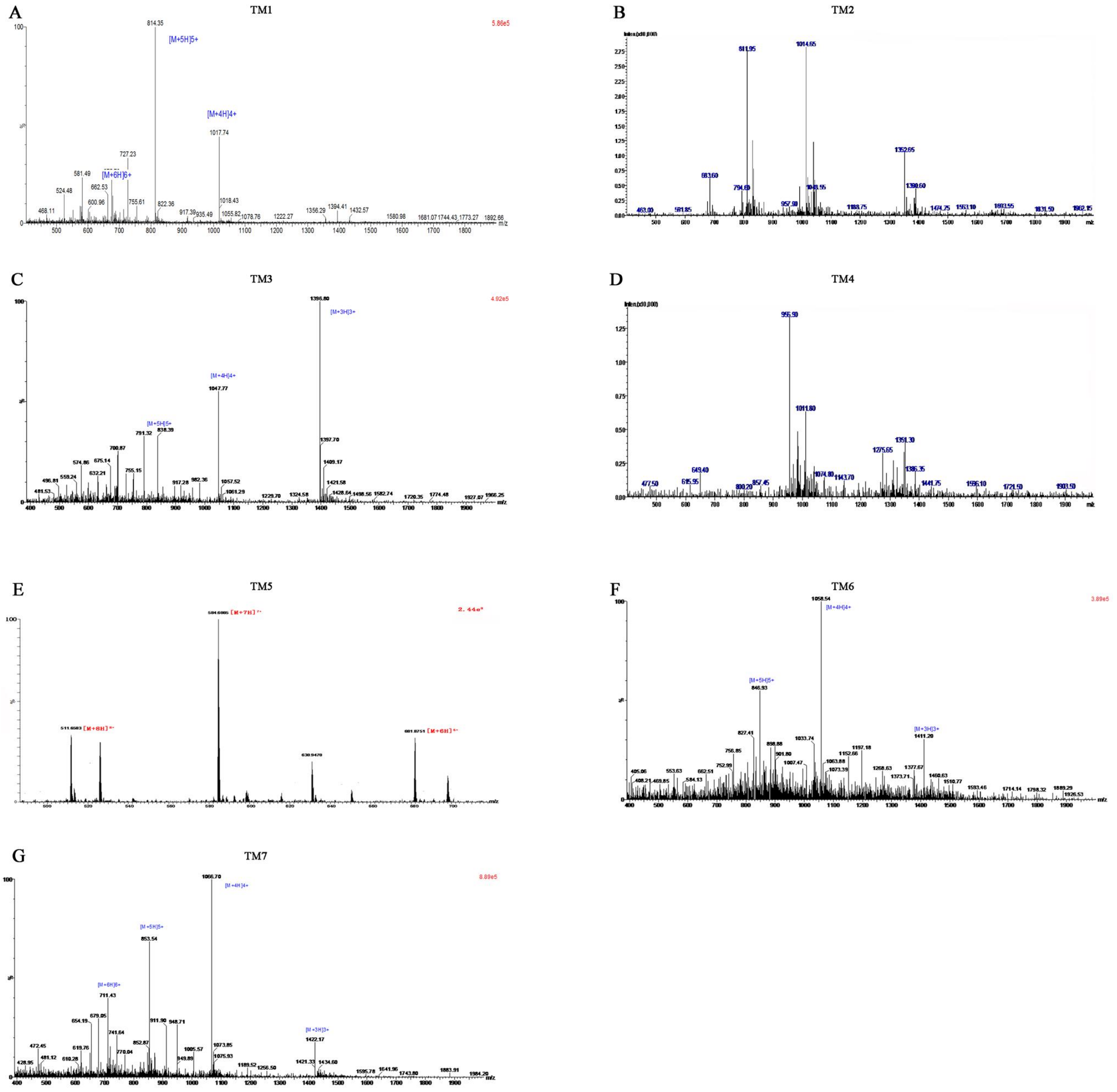
A
TM1
[M+5H]5+
[M+4H]4+
B
TM2
C
TM3
[M+3H]3+
[M+4H]4+
D
TM4
E
TM5
F
TM6
[M+4H]4+
[M+5H]5+
[M+3H]3+
G
TM7
[M+4H]4+
[M+5H]5+
[M+6H]6+
[M+3H]3+

Figure S4

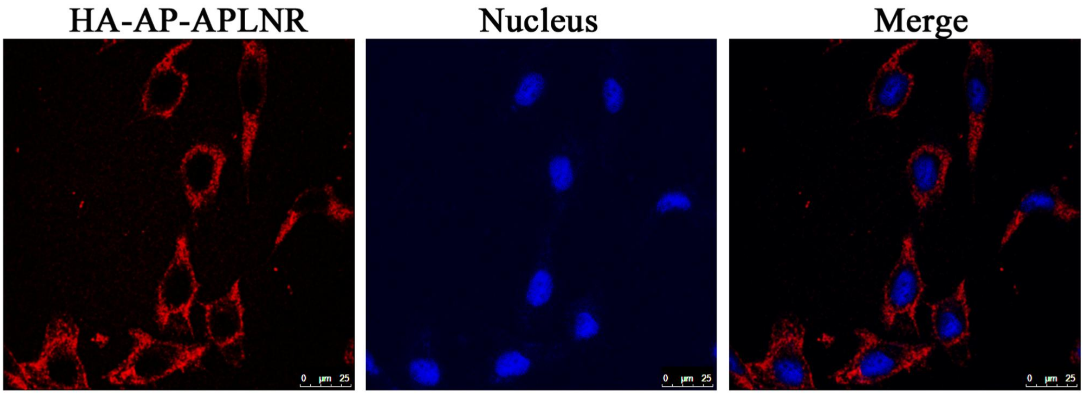

Figure S5

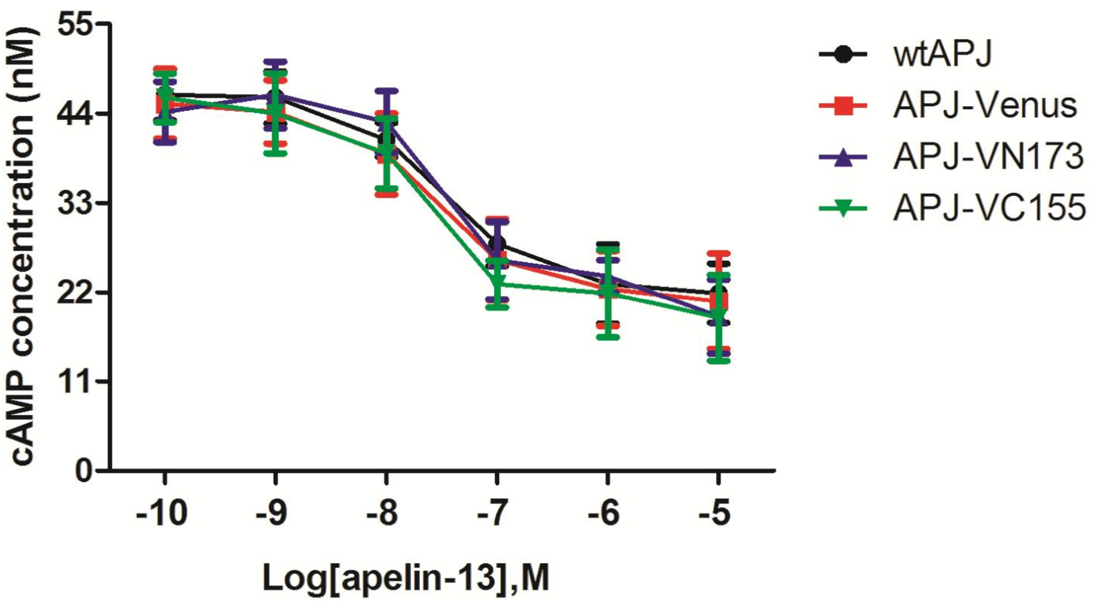
cAMP concentration (nM)
55
44
33
22
11
0
-10
-9
-8
-7
-6
-5
Log[apelin-13],M
wtAPJ
APJ-Venus
APJ-VN173
APJ-VC155

Table S1 Detection of fluorescent resonance energy transfer sensitized emission (FRET-SE) correction factors

Correction factor	Crosstalk values
α	18%
β	60%
γ	52%
δ	35%
